# Risk prediction models for atherosclerotic cardiovascular disease: A systematic assessment with particular reference to Qatar

**DOI:** 10.5339/qmj.2021.42

**Published:** 2021-09-26

**Authors:** Aziz Sheikh, Ulugbek Nurmatov, Huda Amer Al-Katheeri, Rasmeh Ali Al Huneiti

**Affiliations:** ^1^Usher Institute, University of Edinburgh, Edinburgh, UK E-mail: aziz.sheikh@ed.ac.uk; ^2^School of Medicine, Cardiff University, Cardiff, UK; ^3^Strategic Planning and Performance Department, Ministry of Public Health, State of Qatar; ^4^Healthcare Quality and Patient Safety, Strategic Planning and Performance Department, Ministry of Public Health, State of Qatar

**Keywords:** atherosclerotic cardiovascular disease, risk prediction model, systematic review

## Abstract

Background: Atherosclerotic cardiovascular disease (ASCVD) is a common disease in the State of Qatar and results in considerable morbidity, impairment of quality of life and mortality. The American College of Cardiology/American Heart Association Pooled Cohort Equations (PCE) is currently used in Qatar to identify those at high risk of ASCVD. However, it is unclear if this is the optimal ASCVD risk prediction model for use in Qatar's ethnically diverse population.

Aims: This systematic review aimed to identify, assess the methodological quality of and compare the properties of established ASCVD risk prediction models for the Qatari population.

Methods: Two reviewers performed head-to-head comparisons of established ASCVD risk calculators systematically. Studies were independently screened according to predefined eligibility criteria and critically appraised using Prediction Model Risk Of Bias Assessment Tool. Data were descriptively summarized and narratively synthesized with reporting of key statistical properties of the models.

Results: We identified 20,487 studies, of which 41 studies met our eligibility criteria. We identified 16 unique risk prediction models. Overall, 50% (n = 8) of the risk prediction models were judged to be at low risk of bias. Only 13% of the studies (n = 2) were judged at low risk of bias for applicability, namely, PREDICT and QRISK3.Only the PREDICT risk calculator scored low risk in both domains.

Conclusions: There is no existing ASCVD risk calculator particularly well suited for use in Qatar's ethnically diverse population. Of the available models, PREDICT and QRISK3 appear most appropriate because of their inclusion of ethnicity. In the absence of a locally derived ASCVD for Qatar, there is merit in a formal head-to-head comparison between PCE, which is currently in use, and PREDICT and QRISK3.

## Introduction

Atherosclerotic cardiovascular disease (ASCVD) is common and results in considerable morbidity, impairment of quality of life and mortality.^1,2^ A number of ASCVD prediction models have been developed, which were typically being used to generate risk scores over a 10-year period. Some more recent models have estimated longer-term risks.^3^

The first ASCVD model was the Framingham Risk Score.^4^ Subsequent models have broadened the range of risk factors used to include, for example, social deprivation and ethnicity.^5,6^ Many clinical guidelines have recommended the use of ASCVD prediction models^7^ however, deciding on which model(s) to use is not straightforward, as there is a need to take into account background risk factors and the applicability of the model(s) to the target population. Furthermore, studies have shown that models do not necessarily align in terms of their risk estimation.^8^

Choosing the optimal model is of crucial importance given that ASCVD is very prevalent and the leading cause of mortality in Qatar.^9,10^ Between 2009 and 2019, the number of deaths attributable to ischemic heart disease increased substantially. Based on 2019 United Nation estimates that Qatar has a population of 2.8 million people. Approximately 12% are Qatari and 88% are foreign nationals. These foreign nationals come mainly from Asia, with smaller numbers from Africa and Europe.^11^ Ideally, a risk prediction model would be derived using data from the Qatari population; however, this is not available. Currently, Qatari experts have concluded that the American College of Cardiology (ACC)/American Heart Association (AHA) Pooled Cohort Equations (PCE)^12,13^ should be used to evaluate ASCVD risk.

This study sought to identify, assess the methodological quality of and compare the properties of established ASCVD risk prediction models through head-to-head comparisons of established risk prediction models for use in Qatar.

## Methods

We drew on guidance from the Cochrane Prognosis Methods Group, which produced the CHecklist for critical Appraisal and data extraction for systematic reviews of prediction Modeling Studies (CHARMS).^14^ Detailed methods are available in the protocol for this review from PROSPERO with Registration no. CRD42020176981.

### Search strategy

We built on the search strategies developed by Damen et al.,^15^ and Siontis et al.,^16^ which were modified by adding terms to capture newly developed risk prediction models (such as the PCE and GLOBORISK).^17,18^ A sensitive search strategy was developed, and validated study design filters were applied to retrieve articles from MEDLINE (OVID), Embase (OVID) and CINAHL (Ebscohost) (Appendix 1). Databases were searched from 1 July 2013 to 31 July 2019 with earlier relevant studies being identified as per the studies retrieved by Damen et al.,^12^ and Siontis et al.,^13^ Additional studies were identified by searching references cited by included studies. Unpublished work and research in progress were identified through searches on Google and Google Scholar. All searches were undertaken in English.

### Inclusion criteria

The selection criteria are summarized using the Population, Intervention, Comparator, Outcomes, Study design format.

#### Population

We were interested in identifying models developed for use in patients of any age and in any geographical location for the primary prevention of ASCVD.

#### Interventions of interest

This review focused on identifying ASCVD risk prediction models for use in the general population that had been formally derived and validated.

#### Comparator

Comparisons were made between ASCVD risk prediction models.

#### Outcomes

These focused on the properties of models used to investigate incidence or mortality from individual or composite ASCVD outcomes.

#### Study designs

Retrospective and prospective cohort studies were eligible.

### Study selection

All references were uploaded into the systematic review software DistillerSR. Study titles were independently checked by two reviewers according to the above selection criteria. Studies were eligible if they included at least two models in populations without preexisting cardiovascular disease. Full-text copies of potentially relevant studies were obtained, and their eligibility for inclusion was then independently assessed. Any discrepancies were resolved through discussion, and if necessary, a third reviewer was consulted.

### Quality assessment

Quality assessments were independently carried out on each model by two reviewers using the Prediction Model Risk Of Bias Assessment Tool (PROBAST), which have specifically been developed for assessing the risk of bias (ROB) and the applicability of prediction modeling studies.^19^ The latest version of the model was used, although earlier versions were often referred to for details of the derivation and validation cohort(s).

### Data extraction, analysis, and synthesis

Two reviewers independently extracted data onto a customized data extraction sheet in DistillerSR. Methods for quantitatively synthesizing evidence from prognostic modeling studies have yet to be developed. We therefore undertook a detailed descriptive summary with data tables and narrative synthesis of data.

### Discrimination

For each model, discrimination, defined as the ability of the prediction model to identify individuals who developed the event of interest from those who did not, was measured by recording the area under the receiver operating curve (AUROC or AUC). We used a threshold of >5% for each paired comparison to indicate a significant difference.^15^ The C-statistic, D-statistic, Brier score, *p* values and 95% confidence intervals (95% CIs) were also recorded (key definitions are shown in Appendix 2).^15^

### Calibration

Calibration performance, defined as the agreement between the observed and predicted risks, was established for each study by recording the number of observed and predicted events.

### Risk reclassification

Where a risk prediction model was compared with another model, but an additional predictor had been added, risk reclassification analysis should be performed to quantify how well the new model reclassified the subjects. To report data, we recorded the net reclassification index (NRI) and the absolute net reclassification index. The NRI consists of two components, namely, subjects with events and those without events, and then scores subjects on whether their risk was reclassified as higher or lower or not changed using the new prediction model.^20^

### Outcome selection and optimism bias

Some risk prediction models were developed for one cardiovascular outcome, but were then evaluated for a different outcome, which can introduce bias. We recorded the outcomes originally of interest and the outcomes from the models. Some models included investigated a new risk model. Optimism bias can be a relevant consideration in such cases, as the new models can perform better when initially described than older models, but this may not be applicable in subsequent comparisons.^21^

## Results

### Inclusion of studies

**Figure 1.**
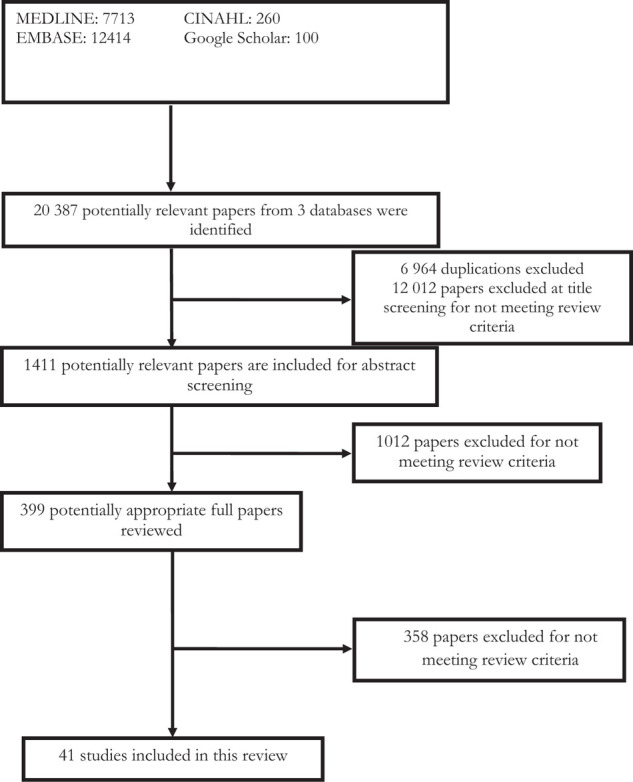
PRISMA flow diagram

We identified 20,487 potentially relevant papers, of which 41 comparative studies satisfied the inclusion criteria (Figure 1).^22–62^

### Characteristics of eligible studies and risk models

The key characteristics of included studies are detailed in Table 1. All included studies employed cohort designs and were published after 2013. Studies were undertaken in Australia (n = 3), China (n = 2), Denmark (n = 2), Germany (n = 2), Bangladesh (n = 1), Finland (n = 1), Hong Kong (n = 1), Iran (n = 1), Israel (n = 1), Italy (n = 1), Japan (n = 1), Malaysia (n = 2), Netherlands (n = 1), New Zealand (n = 1), Peru (n = 1), Portugal (n = 1), South Korea (n = 3), Sweden (n = 1), UK (n = 2) and USA (n = 11). One study from the Netherlands assessed Ghanian migrants,^22^ and another study derived and validated a set of 10-year cardiovascular functions in Spain (FFERSCO Study) with the validation of the Framingham-REGIGOR function.^46^

All included studies had at least one head-to-head comparison between models. Some studies compared several models.^21,22,24,25,28,29,32,34,35,38,39,42,43,47,52,54,55,59^ In total, the 41 studies compared 116 models. These established models included various adaptations of the Framingham equation (n = 39),^63^ Systematic Coronary Risk Estimation (SCORE) calculator (n = 15),^64^ PCE (n = 14),^65,66^ ACC/AHA (n = 7),^64,65^ Adult Treatment Panel (ATP) (n = 4),^64,65^ Reynolds Risk Score (RRS) (n = 4),^67^ World Health Organization (WHO) (n = 4),^68^ Korean Heart Study (n = 2),^37^ Cardiovascular disease risk algorithm (QRISK) (n = 2),^69^ American CVH (n = 1),^70,71^ ssessing cardiovascular risk to Scottish Intercollegiate Guidelines Network (n = 1),^72^ Health 2000 (n = 1),^37^ Finrisk (n = 1),^73^ Korean Risk Prediction Model (KRPM) (n = 1),^39^ Persian Atherosclerotic cardiovascular disease (n = 1),^61^ Cardiovascular Disease Cohort in New Zealand primary care Cohort in New Zealand (PREDICT) (n = 1),^50^ Prospective Cardiovascular Münster score (PROCAM) (n = 1),^74,75^ Suita (n = 1) and ^49^ Veterans Affairs Risk Score – CVD (VARS CVD) (n = 1).^56^

### Risk of bias

We critically appraised each unique risk calculator identified (n = 16) (Table 2 and Appendix 3). This showed that for participant selection, most studies scored at low ROB (n = 12, 75%). The predictors, outcome and analysis were often at low ROB (n = 15, 94%; n = 14, 87%; n = 12, 75%), respectively. The key concern regarding analysis is related to handling of missing data. Only 25% of the studies (n = 4) were at low ROB for participation selection in relation to the applicability concern, as it appeared that the study population did not reflect the Qatari population. All studies were at low ROB for applicability of predictors chosen; 87% (n = 14) of studies were at low ROB for outcome measures in the applicability domain. Overall, 50% (n = 8) of the risk prediction models were judged to be at low ROB. These models were the ACC/AHA (PCE), American CVH, FINRISK, FRS, Health 2000, PREDICT, PROCAM, and RRS. However, only 13% of the studies (n = 2) were judged at low ROB for applicability concern, including QRISK and PREDICT (Table 2). Only the PREDICT risk calculator were at low ROB in both domains.

### Discrimination performance

Of the included studies, at least one measure of predicting performance was reported for 28 of the 41 (68%) models. All these 28 models reported discrimination performance as the AUC.^24,26,28-30,32,34-35,38-45,47-48,50-58,61^

The AUC was reported for men in 15/41 (37%) models,^24,28,35,39,40,42-43,45,50,53-57,61^ for women in 16/41 (39%) models^24,28,32,35,39-40,42-43,45,50,53-57,61^ and overall in 17 of 40 (43%) models^24,26,29,30,34-36,38,40,41,44,47-48,52,54,55,58^ (Supplementary file: Table 3a).

The relative difference between the AUC estimates exceeded 5% in nine of these studies.^29,30,34,54,35,42,45,47,58^ The first of these studies looked at the incidence of stroke (Flueckiger et al., and Foraker at al.,).^29-30^ In the study by Flueckiger et al., both the revised Framingham Stroke Risk Score (FSRS) and the PCE performed better than the old FSRS (0.73, 0.72, and 0.65, respectively).^29^ In the study by Foraker et al., the CVD risk score fared better than the Cardiovascular Health metric (0.79 and 0.59, respectively).^30^ Another two studies focused solely on cardiovascular mortality. In the first study, Harari et al., compared six prediction models, four of which were newly developed.^34^ These four new models performed better than the SCORE High and Low Risk calculators (0.85, 0.85, 0.85, 0.86, 0.81, and 0.79, in this order). In the second study, Selvarajah et al., demonstrated that the FRS, SCORE High and SCORE Low performed better than the WHO/ISH calculator (0.77, 0.77, 0.77, and 0.61, respectively).^54^ In a study of the incidence of coronary heart disease, Hu et al., showed that two new models performed better than six versions of the FRS in male, female and overall population.^35^ In another study, Kavousi et al., compared three different prediction models, two of which (AAA/AHA and ATPIII) focused at predicting cardiovascular events, while the third (SCORE) focused on cardiovascular mortality.^40^ This study found that SCORE performed better than the other two models in both men and women (men, 0.76, 0.67 and 0.67; women, 0.77, 0.68 and 0.69, respectively). In the comparison of the outcome of two studies on predicting cardiovascular events, Lee et al., revealed that FRS demonstrated a better performance than PCE in men alone (0.77 and 0.71, respectively),^45^ and Mortensen et al., 2015 demonstrated that the ACC/AHA model performed better than two trial models (0.68, 0.57 and 0.61, respectively).^47^ The final study by Tralhao et al., examined high coronary atherosclerosis burden and showed that the ASCVD model performed better than SCORE (0.74 and 0.69, respectively).^58^

Discrimination performance according to metrics other than the AUC were reported for three (7%) models: Polypchuk et al., (2018) used D-statistic with 95% CIs for men and women,^50^ Sussman et al., (2017) used Brier score with 95% CIs for men and women,^56^ and Tillin et al., (2014) used D-statistic, R^2^-statistic and Brier score with their 95% CIs for men and women (Supplementary file: Table 3b).^57^ These three studies reported these statistics in addition to AUCs.

AUCs with 95% CIs were given for only 15 (37%) studies.^26,28,30-32,36,39,42-43,45,50,52-55,58,61^ Overall, based on discrimination statistics, compared with other models, the Framingham models were worse in five cases^24,35,38,52,55^ but better in three cases.^29,34,54^ Overall, PCE was worse in one study^29^ and better in three studies.^48,52,55^ SCORE was worse in five studies^34,48,52,55,58^ and better in three studies.^26,48,54^ PREDICT risk score was better than PCE in one study (Supplementary file: Table 3b).^50^

### Calibration

Calibration performance was reported in 21 (51%) studies (Supplementary file: Table 4). Predicted versus observed ratios were reported through Hosmer–Lemeshow Goodness of Fit Chi-squared test,^76, 24,32,35,39,40,45,56^ Nam-D'Agostino Chi-squared or modified Nam-D'Agostino Chi-squared tests, graphical representation of Hosmer–Lemeshow Chi-squared test or calibration plots.^26,29,30-31,34,36,40,42,49,50,53-55,61^ The 95% CIs of the predicted versus observed ratio was available for only one study,^57^ and *p* values were available for 11 studies.^24,29,32,36,39,40,41,53-55,61^ Compared with other models, the FRS was better in two studies for men^45,53^ and worse for one study ^24^; in women, the FRS was better in two studies^24,32^ (Supplementary file: Table 4). Overall, the results of calibration metrics were inconsistent; we could not therefore establish whether these differences were beyond those expected by chance.

### Risk reclassification

Information on risk classification and reclassification was only available for four (10%) studies. In one study,^29^ addition of coronary artery calcium, carotid intima media thickness, C-reactive protein, ankle-brachial index (ABI) and family history of stroke to the revised FSRS showed an improvement in the overall category-less NRI and the Integrated Discrimination Improvement (IDI) (NRI = 0.36, IDI = 0.0027), while ABI demonstrated the least (NRI = 0.11, IDI = 0.0013) improvement. One study reported that a CHD risk model in a Korean population, in comparison with the FRS, employed the basic model using the continuous form of the NRI for the 10-year risk of CHD. From the original derivation model, they created additional three models by adding high-density lipoprotein-cholesterol (Model 1), low-density lipoprotein-cholesterol (Model 2) and triglycerides (Model 3). Model 1 was superior to the original model and had the best reclassification of risk (NRI = 0.284). For women, Model 3 was the best model for reclassification of risk (NRI = 0.177).^37^ The Health 2000 risk score compared with the FRS resulted in a significant NRI (21.7%, *p* < 0.0001).^38^ When chronic kidney disease was included as a predictor into the Suita score in a Japanese population, the NRI was 41.2% (*p* < 0.001) compared with the original FRS.^49^

### Outcome selection bias

In 29 studies,^22-24,26-34,38-48,51-57,59-61^ the outcome of interest was CVD events (fatal and nonfatal). In nine studies, the outcome of interest was CHD-related events^28,32,35-37,42,44,49,51^; in six studies, all-stroke or ischemic stroke incidences were the outcome of interest.^27,29-30,38,42,50^ In one study, the outcome was the agreement with predicted CVD risk using Lin's concordance correlation coefficient.^21^ Another study assessed differences in absolute risk of CV events,^25^ and in one study, the outcome was defined as the comparison of ACC/AHA guidelines to the European Society of Cardiology/European Atherosclerosis Society Guidelines for Primary Prevention of ASCVD for accurately assigning statin therapy to those who would benefit.^48^

### Optimism bias

Twelve (29%) studies described a model for the first time (Appendix 4). In four studies, the new model had a higher AUC estimate compared with the existing models.^38-39,50,61^ Johansson et al., found that the Health 2000 new model had AUC estimate of 0.85 compared with Finrisk (0.84), FRS (0.83) and RRS (0.84).^38^ The KRPM had higher AUC score (0.74) than PCE-white (0.73) and PCE-African-American (0.72).^39^ Interestingly, a new risk prediction model developed in New Zealand (PREDICT) also had higher AUC score (0.73) than PCE (0.71).^50^ Finally, the China-PAR that was derived and validated in China also had higher AUC estimate (0.79 for men and 0.81 for women) than PCE (0.76 for men and 0.78 for women).^61^ However, none of these differences exceeded 5%.

## Discussion

### Principal findings

We systematically examined head-to-head comparisons of established risk prediction models for the primary prevention of ASCVD. Through this process, we identified 41 studies reporting on 16 unique cardiovascular risk prediction models that have been deployed in clinical practice. The majority (54%) of these models were derived from Europe and USA. None had been developed specifically for use in Arab populations. Careful comparisons of these models have shown the lack of overall consistent findings with studies showing in some cases comparable performance and in others superior or inferior performance. In some studies, new models appear to perform better than old established models, but these may be subject to optimism bias, and further studies are needed to verify these results.

### Strengths and limitations

We performed a formal systematic comparison using state-of-the art methods. This study builds on previous work that has been carried out in this field.^14,15^ The newly developed PROBAST tool was used to assess both ROB and applicability of each calculator.^18^

The limitations of this review include the fact that we may not have identified all relevant studies. In some studies, there was poor reporting of data, which made it difficult to assess study quality. Furthermore, as most studies were conducted in Europe or the USA, there were challenges in inferring which risk prediction model(s) would work best for the Qatari population.

### Comparison with other studies

Our results are in keeping with previous systematic reviews.^14,15, 77^ Most reviews concluded that there is now an abundance of cardiovascular risk calculators, but reported difficulty in deciding which is most appropriate to use. Moreover, the majority of the risk calculators have been developed in predominantly White European-origin populations limiting their usefulness for other ethnic groups. The heterogeneity and lack of reporting of discrimination statistics have previously been highlighted^4,15^; for example, we found that 30% of studies reported no statistics whatsoever.

### Implications for policy, practice, and research

We were able to identify models that included ethnically diverse populations in their derivation and

validation cohorts. No model closely resembled Qatar's diverse ethnic profile. That said, the risk

calculators that incorporated ethnicity within their development, i.e. in both the derivation and validation

phases, were PREDICT and QRISK3. Furthermore, using PROBAST to assess ROB and applicability of

each of the individual models, we were able to identify PREDICT and http://chd.bestsciencemedicine.com/calc2.html) and QRISK3 (https://www.qrisk.org/three/) as potential candidates for use in Qatar.^50,69^ Qatar is currently using the ACC/AHA PCE, which although judged to be at a low ROB was not found to be applicable to Qatar's ethnically diverse population. These results were discussed with clinical and policy leaders across Qatar in a workshop and will be used to inform deliberations on the need for formal validation studies in Qatar. These findings may also be applicable to other Arab countries with similar ethnically diverse populations.

## Conclusions

This study commissioned by the Qatari Ministry of Public Health has shown that there is no existing ASCVD risk calculator particularly well suited for use in the ethnically diverse Qatari population. Of the available risk calculators, PREDICT and QRISK3 appear to be best suited for use in Qatar because of their inclusion of ethnicity. In the absence of a locally derived ASCVD for Qatar, there is merit in a formal head-to-head comparison between the currently used PCE, and PREDICT and QRISK3.

### Registration

This systematic review is registered with PROSPERO (Registration no. CRD4202017698).

### Funding

This study was funded by the Qatar Ministry of Public Health.

### Conflicts of interest

AS was involved in the development of the QRISK2 algorithm.

### Acknowledgments

We wish to express our thanks to Dr. Rasmeh Ali Al Huneiti and the lead clinicians and policy leads who contributed to the workshop.

## Figures and Tables

**Table 1:** Characteristics of the included studies

Study	Year	Prediction horizon (years)	Study Design	Study population	Sample size (men/women)	Models	Outcomes	No of events (men/women)

Bazo-Alvares, Peru	2015	10	Cohort	Peru: sites: Ayacucho (n = 83); Lima (n = 871); Puno (Rural)(n = 356); Puno (Urban) (n = 366); and Tumbes (n = 495) age 45–54 (52.9%) and 55–65 (47.4%)	2,183 48.1% male; 51.9% female;	PCE was compared with six other models: 1. FRS, Global CVD: two versions used, FRSlipids and FRS-nonlab 2. ACC/AHA model 3. WHO Risk Chart (WHO/ISH) 4. RRS 5. SCORE project risk score, four versions used: High-risk and low-risk countries, with and without HDL-C 6. Risk Chart developed by LCD group	in agreement with the predicted CVD risk using Lin’s concordance correlation coefficient	N/R

Boateng, The Netherlands	2018	10	Cohort	3586 participants aged 40–70 years in the multicenter RODAM (Research on Obesity and Diabetes among African Migrants) study among Ghanaians residing in Ghana and Europe	3,586 All male (Ghana = 1564; Europe = 2022)	FRS lab FRS nonlab PCE algorithms.	Not specified; 10-year CVD risk. Participants were classified as low, moderate, or high risk, corresponding to < 10%, 10%–20%, and >20%, respectively	Not reported

Chia, Malaysia	2014	10	Retrospective cohort	Asian population: Chinese, Indian, and Malay (mean age: 57.5 ± 8.8 years; 47 diabetic patients; 59.9 antihypertensive treatment	922 66.7% female	PCE FRS General Cardiovascular Disease (CVD) risk score.	nonfatal myocardial infarction, coronary heart disease (CHD) death, fatal and nonfatal stroke	CVD events occurred in 45 of 922 patients, 22 (7.2%) in males and 23 (3.7%) in females

DeFilippis, USA	2015	10.2	Cohort	MESA (Multi-Ethnic Study of Atherosclerosis), a community-based, sex-balanced, multiethnic cohort (mean age, 61.5 years; women, 53.5%; white, 42%; African American, 26%; Hispanic, 20%; and Chinese, 12%)	4,227 (1961 men, 2266 women)	Five risk scores: 1. FRS-CHD 2. FRS-CVD 3. ATP-FRS-CHD 4. RRS 5. AHA/ACC/ASCVD	Observed and expected ASCVD events	Not reported

DeGoma, USA	2013	10	Cohort	1. Framingham (Wilson et al., 1998) (mean age 49; women 53%; white 100%) 2. Women’s Health Study (Ridker et al., 2007) (mean age 52; women 100%; white 95%) 3. Physician’s Health Study (Ridker et al., 2008b)(mean age 63; women 0%; white N/A) 4. ARIC (Nambi et al., 2010) (mean age 54;women 57%; white 75%) 5. MESA (Goff et al., 2006)(mean age 62; women 53%; white 38%)	1. FRS (Wilson et al., 1998) (n = 5,345) 2. Women’s Health Study (Ridker et al., 2007) (n = 16,400) 3. Physician’s Health Study (Ridker et al., 2008b) (n = 10,724) 4. ARIC (Nambi et al., 2010) (n = 13,145) 5. MESA (Goff et al., 2006) (n = 6,704)	FRS risk score, equations derived from the Multi-Ethnic Study of Atherosclerosis (MESA) and the Atherosclerosis Risk in Communities (ARIC) study, and the Reynolds risk score	Differences in absolute 10-year cardiovascular risk between alternative risk equations. Absolute 10-year cardiovascular risk of having CHD for Framingham, MESA, and ARIC risk equations. The Reynolds score, CHD events are included, additionally non CHD death and coronary revascularization, and ischemic stroke. Differences in absolute risk of CV events.	Not reported

De las Heras Gala, Germany	2016	10	Cohort	The Monitoring of Trends and Determinants in Cardiovascular Disease (MONICA)/Cooperative Health Research in the Region of Augsburg (KORA) and the Heinz Nixdorf Recall	9,446 48% men	the ACC/AHA risk score vs. ESC SCORE	nonfatal or fatal ASCVD events	KORA s3 and S4 = 383 (7.8%) incident of ASCVD events HNR = 271 (6.4%) first incident ASCVD events

				(HNR) Studies. The KORA S3 (1994–1995) and S4 (1999–2001), total = 5,238; men, 49%; age 40–79 years old HNR = 4,208				

Dufouil, USA	2017	5 and 10	Cohort	FHS, REGARDS, and 3C cohorts 1. the FHS: the old FSRP (n = 5,734, age = 55); the contemporary epoch (n = 5.072, age = 55) 2. REGARDS (the REasons for Geographic and Ethnic Differences in Stroke) 30,239 participants’ = 45 years of age; 42%	43,574 45% men and 55% women	FSRP old FSRP new	all-stroke and ischemic stroke	In the new FHS, over 10 years of follow-up, there were a total of 277 incident strokes (247 ischemic strokes (IS)), 144 (129 IS) in women.

				black and 58% white for 5-year follow-up. 3. the 3C study: three French cities (Bordeaux, Dijon, and Montpellier) n = 7601, at least 65 years old				

Fatema, Bangladesh	2016	2.5	Cohort	Rural Bangladeshi population The original cohort was initiated in 2008 - the “BADAS-ORBIS Eye Care Project” a rural Bangladeshi population (52989 cohort and 439 subcohort participants) mean age 53.73 ± 10.71 years	52,989 followed up until Aug 2014–Dec 2014 (participation rate, 85.02%) 439 followed up until Aug 2014–Dec 2014 (participation rate, 77.97%)	Model 1 FRS (Laboratory based) Model 2 (WHO with cholesterol) Model 3 (WHO without cholesterol) Model 4 FRS (Nonlaboratory based)	CHD (e.g., MI evidenced from ECG, CV death)	Disease free = 394; total cases = 60 (MI)

Flueckiger, USA	2018	10.7	Cohort	MESA (Multi-Ethnic Study of Atherosclerosis) The mean age was 62 ± 10 years. Thirteen percent had diabetes, 15% were on statin therapy, 20% on aspirin therapy, and 33% on antihypertensives.	6,712 53% female	The revised (R-FSRS), original FSRS, and the PCE	Stroke prediction. Stroke was defined as fatal/nonfatal strokes (hemorrhagic or ischemic).	231 stroke events [182 hemorrhagic (3.4%); 49 ischemic strokes (2.7%)]

Foraker, USA	2016	10	Cohort	The Jackson Heart Study; n = 4,140 5301 participants aged 21–95 years (median	4,140	CVD risk score vs. CVH metric (the American Heart Association/American Stroke	The cumulative incidence of stroke; HR and 95% CIs for stroke within African	Within 10 years, 112 strokes occurred

				54.5; 65% females) between 2000 and 2004, without prior history of stroke (5301); missing data (927); finally included (4140)		association	Americans	

Fox, USA	2016	10	Cohort	The Jackson Heart Study (JHS) External validated cohorts ARIC and MESA	3,689 participants	ACC/AHA CVD risk algorithm vs. FRS	Incident CVD event was defined as the first occurrence of myocardial infarction, coronary heart disease death, congestive heart failure, stroke, incident angina, or intermittent claudication	270 CVD events (166 women)

Goh, Australia 4385	2014 a	10	Cohort	The National Heart Foundation third Risk Factor Prevalence Study: 4487	4,487 Australian women; age between 20–69 years; Mean 42.8 ± 13.2 years	FRS SCORE risk charts for low-risk regions SCORE risk charts for high-risk regions in Europe	Death (all cause); CVD deaths	death (all cause) = 152; CVD deaths = 28

Goh, Australia 5780	2014 b	10	Cohort	The third Australian Risk Factor Prevalence Study (north and south Sydney, Melbourne, Brisbane, Adelaide, Perth, Hobart, Darwin, and Canberra)	4,354 females aged 20–69 years	the FRS model vs. SCORE	CHD and CVD incidence and/or mortality	

Harari, Israel	2017	10 and 20	Cohort	A longitudinal Israeli industrial cohort (CORDIS cohort)	4,809 all male	FHS SCORE modified FHS model (FHS/Cox) Omnibus/Cox	CV mortality	76 CVD mortality events during the 10-year follow-up period and 170 during the 20-year follow-up period

Hu, USA	2016	ARIC 10 years NHANES 14 years	Cohort	1. ARIC (the Atherosclerosis Risk in Communities): 13,657 participants, aged 45–64 years 2. NHANES III n = 5,706; 40–70 years old individuals	19,363 males and females	1. FRSv1 from 1998 2. FRSv2 from National Cholesterol Education Program Adult Treatment Panel III, 3. FRSHDL in which the high-density lipoprotein (HDL) component of FRSv1 was ignored 4. NEW CHD with a new-CHD model	MI, fatal CHD, CHD-associated death	ARIC = 13,657; 759 CHD cases, NHANES III n = 5,706; 88 CHD-associated deaths

Hua, Australia	2017	5 and 10 years Maximal follow-up time of 10.5 and 16.4 years	Cohort	The Well Person’s Health Check Indigenous cohort 1448 (women = 748; mean age 45.2 ± 11.6; men = 700, mean age 44.9 ± 11.0) Aboriginal and Torres Strait Islanders from remote Indigenous communities in Far North Queensland.	1,448 (748 women; 700 men)	FRS 1991; FRS 2008; Recalibrated FRS 2008	CHD (including myocardial infarction, angina Pectoris, and coronary insufficiency), CHD death, stroke, congestive heart failure and peripheral vascular disease	369 CVD events (25.5%)

Jee, Korea	2014	11.6	Cohort Prospective	The Korean Heart Study (KHS)	268,315 164,005 men, 104310 women Koreans aged 30–74 years	Develop a CHD risk model among the Korean Heart Study (KHS) population and compare it with the Framingham CHD risk score	Nonfatal or fatal CHD events between 1997 and 2011.	2596 CHD events (1903 nonfatal (MI) and 693 fatal (e.g., sudden death)

Johansson, Finland	2016	11.2	Cohort	The Health 2000 Study from Finland between autumn 2000 to spring 2001; mean age 51.9 ± 14.5	5,843 44.8% men	Health 2000; Finrisk, Framingham, and RRS	Cardiovascular mortality, nonfatal myocardial infarction, nonfatal stroke, percutaneous coronary intervention, and coronary artery bypass surgery.	CV event = 557 No CV event = 5286

Jung, Korea	2015	10	Cohort	The KHS (the Korean Heart Study): aged 40–79 years (1996–2001); Men = 119,715 (mean age 50.13 ± 7.94); (mean age 51.81 ± 8.12)	200,010 adults 80,293 women 119,717 men	PCE vs. a KRPM	First “hard” ASCVD events, comprising the occurrence of death from CHD or fatal stroke or the first occurrence of nonfatal MI or stroke	12,327 ASCVD events (2175 nonfatal MI, 478 fatal CHD, 10,049 nonfatal stroke, and 749 fatal stroke)

Kariuki, USA	2017	12	Cohort	Atherosclerosis Risk in Communities (ARIC) dataset: Secondary analysis. 11,601 participants (mean age, 54 years; 23% black)	11,601 55% women	The nonlab FRS algorithm vs. the lab-based FRS algorithm	CVD events (coronary death, myocardial infarction, coronary artery bypass grafting, and percutaneous coronary intervention), cerebrovascular events (i.e., ischemic stroke, hemorrhagic	1545 new cases of CVD

							stroke, and transient ischemic attack), and heart failure.	

Karjalainen, Sweden	2017	10	Cohort	The Northern Sweden MONICA 2014 cohort: 40–65 years	813 53% women	2003 SCORE Sweden vs. 2015 SCORE Sweden	The high and very high-risk group for cardiovascular death	2015 SCORE Sweden observed CVD death 34 vs. predicted 44.3 2003 SCORE Sweden observed CVD death 34 vs predicted 77.3

Kavousi, The Netherlands	2014	10	Cohort	Rotterdam Study participants aged 55 years or older in the Ommoord district of Rotterdam.	4,854 aged 55 years or older, mean age of participants was 65.5(SD, 5.2) years, and 54.5% were women.	Guidelines: ACC/AHA; ATP-III; and ESC	“hard” ASCVD events (including fatal and nonfatal CHD and stroke) (ACC/AHA), hard CHD events (fatal and nonfatal MI, CHD mortality) (ATP-III), and the ASCVD mortality (ESC).	1. ACC/AHA: hard ASCVD 343; men = 192; women = 151 2. ATP-III: 160; men = 98; women = 62; 3. ESC: CVD death = 87 (men = 50; women = 37

Kempf, Germany	2016	10	Cohort	The Boehringer Ingelheim cohort 4,005 participants; mean age 46.7 ± 5.8; men (54%) 134 men (6%) and 111 women (6%) had current CVD	4,005	FRS, PRS, RRS	10-year CVD risk estimation	10-year CVD risks of 35% (FRS), 9% (PRS), and 6% (RRS) for men and 10% (FRS), 4% (PRS), and 1% (RRS) for women

Lee, Korea	2014	10	Cohort	KNHANES: 7,594 Korean adults, aged 40–79 years;	7,594 male = 3307, Women = 4287	PCE vs. ATP-III equation	ASCVD risk and CHD risk; Also lipid management eligibility	N/R

Lee, Hong Kong	2015		Cohort	The Hong Kong Cardiovascular Risk Factor Prevalence Study (CRISPS): aged 25–74 years	2,895 Chinese men and women	PCE vs. FRS CV risk estimation	CV event, CHD, stroke, all fatal and nonfatal events (MI)	PCE of 1476 subjects aged 40–79, 122 developed hard ASCVD (80 men, 42 women) The

								Framingham CV risk equation of 1668 subjects aged 30–74 years, 138 developed CVD (86 men, 52 women)

Marrugat, Spain	2014	10	Cohort	The FRESCO study (Función de Riesgo ESpañola de acontecimientos Coronarios y Otros, or “Spanish risk function of coronary and other cardiovascular events”) 11 population cohorts in 7 Spanish regions examined between 1992 and 2005: CORSAIB; DRECA-2; MURCIA; EMMA; REGICOR; REUS; ZONA FRANCA; NAVARRA and RIVANA; TALAVERA; and ZACARIS cohorts.	50,408 participants in total aged between 35 and 79 years 23,289 (46.2%) men and 27,119 (53.8%) women	A set of 10-year cardiovascular risk predictive functions in Spain vs. Framingham-REGIGOR	CV events (fatal and nonfatal CHD, stroke, fatal events, MI, angina, other CV)	2973 cardiovascular events Fatal events (n = 2,301) at least one nonfatal CV event (n = 2500)

Mortensen, Denmark	2015	10	Cohort	CGPS (Copenhagen General Population Study): (48–64 years);	37,892 57% women	Risk-based approach - ACC/AHA Guidelines vs. Trial-based	ASCVD events, MI	ASCVD events = 834; MI = 323

						approach - Ridker et al. vs. Hybrid approach - Ridker et al.		

Mortensen, Denmark	2017	5–10	Cohort	44,889 individuals aged 40–75 recruited in 2003–2009 in the Copenhagen General Population Study; all free of ASCVD, diabetes, and statin use at baseline	44,889 men = 19383 women = 25506	USPCE for any ASCVD European-SCORE for fatal ASCVD	Comparison of ACC/AHA guidelines to the ESC/EAS guidelines for primary prevention of ASCVD, for accurately assigning statin therapy	2217 any ASCVD events and 199 fatal ASCVD events through 2014

Nishimura, Japan	2014	11.8	Cohort	Urban population; The Suita cohort study started in 1989	5,521 (male 2,796; mean age 56.1 ± 13.3 and female 2,725; mean age 54.5 ± 12.9	The Suita Score compared with the FRS (the original and the calibrated)	The incidence of CHD; MI; sudden cardiac death	213 cases of CHD

Pylypchuk, New Zealand	2018	Mean follow-up 4.2 years, a third of participants were followed for 5 years or more.	Cohort prospective	Primary Care; 401,752 people between Aug 27, and Oct 12, 2015 European, Indian, Maori, Chinese, or other Asian	401,752 226,053 men and 175,699 women aged 30–74 years (mean age (SD) women = 56 (8.9); Men = 51.8 (9.9)	New PREDICT absolute risk prediction equations vs. ACC/AHA Pooled-Cohort Equations	IHD (including angina); ischemic or hemorrhagic cerebrovascular events (including transient ischemic attacks); or peripheral vascular disease, congestive heart failure, or other ischemic cardiovascular disease deaths	15,386 (4%) participants had CVD events (1507 [10%] were fatal, and 8,549 [56%] met the PCEs definition of hard ASCVD during 1,685,521 person-years follow-up.

Pursnani, USA	2015	Median follow-up 9.4 years (interquartile range 8.1–10.1)	Cohort	Third generation cohorts of the Framingham Heart Study; Framingham multidetector computed tomography (MDCT) imaging study (2002–2005)	2,435 participants, mean age 51.3 (SD, 8.6), 56 women and the mean FRS 6.7%	The ACC/AHA guidelines vs. the National Cholesterol Education Program’s 2004 Updated Third Report of the Expert Panel on Detection, Evaluation, and Treatment of High Blood Cholesterol in Adults (ATP-III) guidelines	The primary outcome was incident CVD (MI, death due to CHD, or ischemic stroke). Secondary outcomes were CHD and CAC (as measured by the Agatston score)	74 incident CVD events (40 nonfatal myocardial infarctions, 31 nonfatal ischemic strokes, and 3 fatal CHD events)

Qureshi, USA	2016	10	Cohort	MESA; 47.1% men; 37.1% whites; 27.2% blacks; 22.3% Hispanics; 12.0% Chinese-Americans	5,654 mean age 61.4 years, 47% male and 53% female	PCE criterion Modified FRS SCORE	Incident ASCVD, composed of fatal and nonfatal myocardial infarction, other fatal and nonfatal coronary heart disease, fatal and nonfatal cerebrovascular disease, and fatal/nonfatal other atherosclerotic disease.	642 (6%) incident AVCD events

Sarrafzadegan, Iran	2017	10	Cohort	The Isfahan Cohort study (ICS)	5,432 participants (Average age for men and women was 51.2 ± 11.9 and 50.3 ± 11.3 years, respectively; 2784 women, 51.3%)	Gender-specific PARS risk chart compared with the Framingham model	The risk of ischemic CVD events, including sudden cardiac death due to unstable angina, myocardial infarction, and stroke	705 events (564 IHDs, 141 strokes)

Selvarajah, Malaysia	2014	5	Cohort	The 2006 National Health and Morbidity Survey dataset; 14,863 participants aged 40–60 years	14,863 participants aged 40–60 years (mean 50.4) male = 45.3% women = 54.7%	The FRS, SCORE WHO/ISH models	CV mortality	5-year mortality 1% (n = 148; 98 = men; 50 = women)

				(mean 50.4 (7))				

Sun, China	2017	6	Cohort	Chinese rural population of Henan Province, China aged 40–65 years	10,338 male = 3945 (38.16) female = 6393 (61.84)	General-FRS, simplified-FRS, SCORE-high, SCORE-low, CN-ICVD, PCE-white PCE-AA were assessed and recalibrated	CVD deaths	168 CVD deaths

Sussman, USA	2017	5	Cohort	VA ambulatory care services EHR data: aged 45–80 years old (mean 61.7 ± 8.6); 95% male at specific ambulatory care clinics in 2006.	1,512,092 patients (1,435,937 men; 76,155 men)	VA Risk Score-CVD (VARS-CVD) and ASCVD score. Population Recalibrated, Regression Recalibrated	Hard CVD events: the first occurrence of nonfatal myocardial infarction, CHD death, fatal or nonfatal stroke	N[R

Tillin, UK	2014	Baseline 1988–91 Follow-up 2008–11	Cohort prospective	SABRE 1866 white Europeans, 1377 South Asians, and 578 African Caribbeans,	4,539 at baseline, 4228 at follow-up, 3821 had follow-up data, aged 40–69 at baseline men and women	QRISK2 vs. Framingham 3 with South Asian adjustment	First CVD events: myocardial infarction, coronary revascularization, angina, transient ischemic attack or stroke reported by participant, primary care or hospital records or death certificate	Follow-up data were available for 3821 (90%). 387 (14%) CVD events occurred in men and 78 in women (8%); 82% of these were CHD events

Tralhao, Portugal	2016	N/A	Cohort	Single-center prospective registry of patients undergoing coronary computed tomography angiography, aged 40–75 years without diabetes or known cardiovascular disease.	327 patients assessed (181 men,146 women) mean age 59 ± 9 years	PCE SCORE	High coronary atherosclerosis burden; coronary artery calcium score (CACS)	45% no visible coronary calcification; only 27 (8%) had a CACS greater than 400. CACS was quantified by the Agatston method and a CACS cutoff value of greater than or equal to 300 was used to define severe

								coronary artery calcification

Van Staa, UK	2014	10	EHR Cohort	Using the November 2011 version of CPRD and drawn from CPRD practices in the UK GP that participated in the linkages	1.8 million aged 35–74 years	FRS ASSIGN QRISK2	CVD as recorded by the GPs (MI, angina, CHD, stroke, and transient ischemic attack). Hospitalization due to CVD Death due to CVD	CVD outcomes occurred in 69,870 persons

Veronesi, Italy	2017	16	Cohort	Data from seven cohorts recruited between 1986 and 1996 in Brianza and in Latina (the MATISS study). All cohorts took part in the collaborative CUORE Project	8,328 3,935 men; 4393 women	“combined” (SCORE R CAMUNI-MATISS) vs “current” SCORE alone stratification	Major CVD event, fatal or nonfatal	CVD events: 468 in men and 210 in women

Yang, China	2016	10	Cohort	Derivation cohort: two cohorts): The China-PAR project used InterASIA and China MUCA (1998)) to develop the Chinese ASCVD risk equations Validation cohort from two independent cohorts MUCA (1992–1994) and CIMIC with 14,123 and 70,838 participants.	21,320 35–74 years old	The China-PAR project compared with PCE	Incident ASCVD events	Derivation cohort: 21320; average follow-up of 12.3 years, 1048 incident ASCVD events (645 in men and 403 in women) were identified


Key ASSIGN: assessing cardiovascular risk to Scottish Intercollegiate Guidelines Network to assign preventative treatment; CABG: coronary artery bypass graft; China MUCA (1998): China Multi-Center Collaborative Study of Cardiovascular Epidemiology; China-PAR project: Prediction for ASCVD Risk in China; CN-ICVD: Chinese ischemic CVD; CI: Confidence intervals; CPRD: Clinical Practice Research Datalink; CIMIC: Community Intervention of Metabolic Syndrome in China &Chinese Family Health Study; CN-ICVD: Chinese ischemic CVD; CVD: Cardiovascular disease; EHR: Electronic Health Record; ESC SCORE: European Society of Cardiology risk score; FRS: Framingham Risk Score; FRSP old: Framingham Stroke Risk Profile; FRSP new: Framingham Stroke Risk Profile new; gen-FRS: general Framingham risk score; GFRS: Global Framingham risk score; sim-FRS: simplified Framingham risk score; IHD: ischemic heart disease; InterASIA: International Collaborative Study of Cardiovascular Disease in Asia; KRPM: Korean Risk Prediction Model; LCD: Lancet Chronic Disease; MATISS: Malattia ATerosclerotica Istituto Superiore di Sanita` study; MESA: Multi-Ethnic Study of Atherosclerosis; MI: myocardial infarction; MONICA-Brianza and the PAMELA: Pressioni Arteriose Monitorate e Loro Associazioni studies; NA: not applicable; NI: Northern Ireland cohort.; PCE: Pooled-Cohort Equation [atherosclerotic cardiovascular disease (ASCVD) score]; PCE-AA: Pooled-Cohort Equations African American; PCE-white: Pooled-Cohort Equations White; PROCAM: Prospective Cardiovascular Münster score; PTCA: percutaneous transluminal coronary angioplasty; SABRE: Southall And Brent REvisited cohort; SCORE: systematic coronary risk evaluation; SCORE-high: High-risk systematic coronary risk evaluation; SCORE-low: Low-risk systematic coronary risk evaluation; Suita Score: Coronary prediction algorithms for Japanese; VA: Veterans Affairs; WHO/ISH: WHO/International Society of Hypertension

**Table 2:** Critical appraisal of selected prediction modeling studies based on the PROBAST checklist (n = 16)

	Risk of bias				Applicability			Overall	

Risk calculator	Participant selection	Predictors	Outcome	Analysis	Participant selection	Predictors	Outcome	Risk of Bias	Applicability

ACC/AHA (PCE)	+	+	+	+	-	+	+	+	-

American CVH	+	+	+	+	-	+	+	+	-

ASSIGN	?	+	+	+	?	+	+	?	?

ESC/SCORE	+	+	+	?	?	+	+	?	?

FINRISK	+	+	+	+	?	+	+	+	?

Framingham	+	+	+	+	?	+	+	+	?

Health 2000	+	+	+	+	?	+	+	+	?

Korean Heart study	-	+	+	-	-	+	+	-	-

Korean Risk Prediction model	?	+	+	-	?	+	+	-	?

PARS	+	+	?	+	+	+	?	?	?

PREDICT	+	+	+	+	+	+	+	+	+

PROCAM	+	+	+	+	?	+	+	+	?

QRISK 3	+	+	+	?	+	+	+	?	+

Reynolds	+	+	+	+	?	+	+	+	?

SUITA	?	+	+	+	?	+	+	?	?

VARS CV	+	?	?	+	+	+	?	?	?


(+) = low risk of bias or applicability concern, (?) = unclear risk of bias or applicability concern, (-) = high risk of bias or applicability concern PROBAST Prediction model Risk of Bias ASsessment Tool

**Table 3a:** Discrimination performance according to area under the receiver operating characteristic curve (AUC) metric (n=41)

Study	Year	Outcome	Model	AUC (95% CI)		
				Men	Women	Overall
Bazo-Alvares	2015	In agreement with predicted	FRS, Global CVD	No data	No data	No data
et al.		CVD risk using Lin’s	ACC/AHA	No data	No data	No data
		concordance correlation	WHO/ISH	No data	No data	No data
		coefficient	RRS	No data	No data	No data
			SCORE	No data	No data	No data
			Risk chart developed by the LCD Group	No data	No data	No data
Boateng et al.	2018	10-year CVD risks	Framingham laboratory	No data	No data	No data
			Framingham non-laboratory	No data	No data	No data
			PCE	No data	No data	No data
			Pooled cohort risk score	No data	No data	No data
Chia et al.	2014	(nonfatal MI, CHD, death, fatal	Framingham general CVD risk score	No data	No data	No data
		and nonfatal stroke)	Pooled cohort risk score	No data	No data	0.632 [0.557, 0.70] P <0.003
DeFilippis et al.	2015	ASCVD events	FRS-CHD	0.69	0.60	0.68
			FRS-CVD	0.71	0.70	0.71
			ATP III-FRS-CHD	0.71	0.67	0.71
			RRS	0.70	0.72	0.72
			AHA/ACC/ASCVD	0.71	0.70	0.71
DeGoma et al.	2013	Differences in absolute risk of	FRS	No data	No data	No data
		CV events	MESA	No data	No data	No data
			ARIC	No data	No data	No data
			RRS	No data	No data	No data
De las Heras Gala	2016	Non-fatal or fatal ASCVD	ACC/AHA	No data	No data	0.76 [0.73, 0.79]
et al.		events	ESC SCORE	No data	No data	0.81 [0.75, 0.85]
Dufouil et al.	2017	All-stroke and ischaemic stroke	Old FSRP	No data	No data	No data
			New FSRP	No data	No data	No data
Fatema et al.	2016	CHD (e.g. MI, CV death)	Model 1 FRS (laboratory based)	0.67 [0.57, 0.77]	0.63 [0.53, 0.71]	No data
			Model 2 (WHO with cholesterol)	0.64 [0.54, 0.75]	0.63 [0.52, 0.73]	No data
			Model 3 (WHO without cholesterol)	0.63 [0.53, 0.73]	0.61 [0.51, 0.72]	No data
			Model 4 FRS (non-laboratory based)	0.63, [0.52, 0.73]	0.69 [0.58, 0.79]	No data
Flueckiger et al.	2018	Incident stroke	R-FSRS	No data	No data	0.73
			O-RSRS	No data	No data	0.65
			PCE	No data	No data	0.72
Foraker et al.	2016	Incident stroke	CVD risk score	No data	No data	0.79 [0.76, 0.83]
			CVH metric	No data	No data	0.59 [0.55, 0.64]
Fox et al.	2016	Incident CVD event	ACC/AHA CVD risk algorithm	No data	No data	No data
			FHS	No data	No data	No data
Goh et al.	2014 a	CHD and CVD incidence and/or mortality	Framingham risk score	No data	0.85 [0.79, 0.93]	No data
			SCORE-Low risk score	No data	0.88 [0.83, 0.93]	No data
			SCORE-High risk score	No data	0.88 [0.83, 0.93]	No data
Goh et al.	2014 b	CVD incidence/mortality	FRS	No data	No data	No data
			SCORE	No data	No data	No data
Harari et al.	2017	Cardiovascular mortality	SCORE-High risk	No data	No data	0.81
			SCORE-Low risk	No data	No data	0.79
			10-year risk FHS/Cox	No data	No data	0.85
			20-year risk FHS/Cox	No data	No data	0.85
			10-year risk Omnibus/Cox	No data	No data	0.85
			20-year risk Omnibus/Cox	No data	No data	0.86
Hu et al.	2016	CHD (fatal and non-fatal)	New-CHD model - NHNES	0.73	0.78	0.75
			New-CHD model - ARIC	0.69	0.76	0.76
			FRSv2 - NHNES	0.66	0.73	0.71
			FRSv2 - ARIC	0.61	0.69	0.72
			FRSv1 - NHNES	0.67	0.74	0.72
			FRSv1 - ARIC	0.66	0.71	0.67
			FRSHDL - NHNES	0.67	0.77	0.71
			FRSHDL - ARIC	0.57	0.67	0.60
Hua et al.	2017	CHD incidence/mortality	Framingham 1991	No data	No data	0.67 [0.64, 0.70]
			Recalibrated Framingham 2008	No data	No data	0.67 [0.65, 0.70]
Jee et al.	2014	CHD (fatal and non-fatal)	Korean Heart Study	No data	No data	No data
			Framingham CHD risk score			
Johansson et al.	2016	CV mortality, nonfatal MI,	Health 2000	No data	No data	0.85
		stroke, PCI, CABS	Finrisk	No data	No data	0.84
			Framingham	No data	No data	0.83
			Reynolds	No data	No data	0.84
Jung et al.	2015	First “hard” ASCVD events (fatal or non-fatal)	PCE White	0.73 [0.72, 0.73]	0.74 [0.73, 0.75]	No data
			PCE African-American	0.72 [0.72, 0.73	0.74 [0.73, 0.75]	No data
			KRPM	0.74 [0.73, 0.75]	0.74 [0.73, 0.76]	No data
Kariuki et al.	2017	CVD events	Non-LB Framingham	0.67	0.75	0.71
			Laboratory-based Framingham algorithm	0.68	0.76	0.71
Karjalainen et al.	2017	CVD mortality	2015 SCORE Sweden			0.82
			2003 SCORE Sweden			No data
Kavousi et al.	2014	“Hard” ASCVD events (including fatal and nonfatal CHD and	ACC/AHA	0.67 [0.63, 0.71]	0.68 [0.64, 0.73]	No data
		stroke) (ACC/AHA), hard CHD events (fatal and nonfatal MI,	ATP - III	0.67 [0.62, 0.72]	0.69 [0.63, 0.75]	No data
		CHD mortality) (ATP-III) and the ASCVD mortality (ESC)	ESC	0.76 [0.70, 0.82]	0.77 [0.71, 0.83]	No data
Kempf et al.	2016	CVD risk estimation	Framingham	0.62 [0.57, 0.67	0.56 [0.53, 0.62]	No data
			PROCAM	0.59 [0.54, 0.64]	0.54 [0.48, 0.54]	No data
			RRS	0.62 [0.57, 0.67]	0.58 [0.52, 0.63]	No data
Lee et al.	2014	ASCVD risk and CHD risk, lipid	ASCVD risk assessment	No data	No data	0.70
		management eligibility	CHD risk assessment	No data	No data	0.64
Lee et al.	2015	CV events (fatal and non-fatal)	PCE	0.71 [0.57, 0.77]	0.76 [0.69, 0.84]	No data
			Framingham CV risk equation	0.77 [0.74, 0.80]	0.79 [0.72, 0.85]	No data
Marrugat et al.	2014	CV events (fatal and non-fatal)	CVR prediction model (Spain)	No data	No data	No data
			Framingham-REGIGOR	No data	No data	No data
Mortensen et al.	2015	ASCVD events	ACC/AHA approach	No data	No data	0.68
			Trial-based approach	No data	No data	0.57
			Hybrid approach	No data	No data	0.61
Mortensen et al.	2017 ?	Comparison of ACC/AHA	US-PCE	No data	No data	0.71–0.85
		guidelines with the ESC/EAS guidelines for primary prevention of ASCVD, for accurately assigning statin therapy to those who would benefit	SCORE	No data	No data	0.69–0.84
Nishimura et al.	2014	CHD incidence/mortality	FRS and recalibrated FRS	No data	No data	No data
			Coronary prediction algorithm for Japanese	No data	No data	No data
Polypchuk et al.	2018	Ischaemic heart disease events/mortality	PREDICT 1 equations	0.73 [0.72, 0.73]	0.73 [0.72, 0.73]	No data
			PCE	0.71 [0.70, 0.72]	0.71 [0.70, 0.72]	No data
Pursnani et al.	2015	CVD incidence or mortality;	ACC/AHA	No data	No data	No data
		CHD and CAC (as measured by the Agatston score)	ATP III	No data	No data	No data
Qureshi et al.	2016	Incidence CVD (fatal/non-fatal)	PCE	No data	No data	0.74 [0.71, 0.76]
			SCORE (high risk)	No data	No data	0.72 [0.70, 0.75]
			SCORE (low risk)	No data	No data	0.72 [0.70, 0.75]
			FRS	No data	No data	0.72 [0.69, 0.74]
Sarrafzadegan et al.	2017	CVD events/mortality	PARS risk chart	0.73 [0.70, 0.76]	0.75 [0.73, 0.78]	No data
			Framingham	0.76 [0.75, 0.78]	0.79 [0.77, 0.81]	No data
Selvarajah et al.	2014	CV mortality	FRS	0.75 [0.71, 0.79]	0.76 [0.70, 0.81]	0.77 [0.73, 0.80]
			SCORE (high risk)	0.77 [0.73, 0.81]	0.76 [0.71, 0.81]	0.77 [0.74, 0.81]
			SCORE (low risk)	0.77 [0.73, 0.81]	0.76 [0.71, 0.81]	0.77 [0.74, 0.81]
			WHO/ISH	0.62 [0.56, 0.68]	0.60 [0.52, 0.68]	0.61 [0.56, 0.66]
Sun et al.	2017	CV mortality	general-FRS	0.72 [0.70, 0.73]	0.74 [0.73, 0.75]	0.73 [0.72, 0.74]
			simplified-FRS	0.71 [0.70, 0.73]	0.75 [0.74, 0.76]	0.73 [0.73, 0.74]
			SCORE-low	0.73 [0.72, 0.75]	0.74 [0.73, 0.75]	0.73 [0.72, 0.74]
			SCORE-high	0.73 [0.71, 0.74]	0.74 [0.73, 0.75]	0.73 [0.72, 0.74]
			CN-ICVD	0.73 [0.72, 0.74]	0.73 [0.72, 0.74]	0.73 [0.73, 0.74]
			PCE-white	0.71 [0.70, 0.73]	0.74 [0.73, 0.75]	0.73 [0.72, 0.74]
			PCE- AA	0.74 [0.72, 0.75]	0.74 [0.73, 0.75]	0.74 [0.74, 0.75]
Sussman et al.	2017	Hard CVD events (incidence/	VARS - CVD	0.66	0.73	No data
		mortality)	ASCVD model	0.66	0.73	No data
Tillin et al.	2014	First CVD events	QRISK	0.72	0.74	No data
			FRS	0.72	0.74	No data
Tralhao et al.	2016	High coronary atherosclerosis burden (CACS)	ASCVD calculator	No data	No data	0.74 [0.67, 0.82] 0.72 [0.64, 0.80]
			SCORE	No data	No data	0.69 [0.61, 0.78] 0.68 [0.60, 0.76]
Van Staa et al.	2014	CVD incidence	Framingham	No data	No data	No data
			ASSIGN	No data	No data	No data
			QRISK2	No data	No data	No data
Veronesi et al.	2017	CVD events (fatal; non-fatal)	SCORE	No data	No data	No data
			SCORE+CAMUNI-MATISS	No data	No data	No data
Yang et al.	2016	ASCVD incidence	China-PAR	0.79 [0.77, 0.81]	0.81 [0.79, 0.83]	No data
			PCE	0.76 [0.74, 0.78]	0.78 [0.76, 0.81]	No data

**Key:** *AA, African-American; ACC/AHA, American College of Cardiology/American Heart Association; ARIC, Atherosclerosis Risk in Communities; ASA , American Stroke Association ; ASCVD, Atherosclerotic cardiovascular disease; ASSIGN, assessing cardiovascular risk to Scottish Intercollegiate Guidelines Network to assign preventative treatment score; ATP-III, Adult Treatment Panel III; CABS, coronary artery bypass surgery; CABG, coronary artery bypass graft; CACS, coronary artery calcium score; CAMUNI - MATISS, CArdiovascular Monitoring Unit in Northern Italy - Malattia ATerosclerotica Istituto Superiore di Sanita; CHD, coronary heart disease; CN-ICVD, Chinese ischemic CVD; China-PAR, Prediction for ASCVD Risk in China; CVD, cardiovascular disease; CVH metric, cardiovascular health metric; CVR, cardiovascular risk; ESC, European Society of Cardiology; ESC SCORE, European Society of Cardiology risk score; FHS, Framingham Heart Study; FRS, Framingham risk score; FRSHDL, HDL of FRSv1 was ignored; FSRS, Framingham Stroke Risk Score; FSRP, Framingham Stroke Risk Profile; KRPM, Korean Risk Prediction Model; LCD, Lancet Chronic Disease; MESA, Multi-Ethnic Study of Atherosclerosis; NHNES, National Health and Nutrition Examination Survey; PARS, Persian Atherosclerotic cardiovascular disease; PCE, Pooled Cohort Equations; PCI, percutaneous coronary intervention; PROCAM, Prospective Cardiovascular Münster score; PTCA, percutaneous transluminal coronary angioplasty; QRISK, cardiovascular disease risk algorithm; REGICOR, Registre Gironí del Cor or Girona Heart Registry; RRS, Reynolds Risk Score; SCORE, Systematic Coronary Risk Evaluation; VARS-CVD, Veterans Affairs Risk Score - CVD; WHO/ISH, World Health Organization Risk Chart*

**Table 3b:** Discrimination performance according to metrics other than the AUC

First author	Model	D statistic (95% CI)			R^2^ statistic (95% CI)			Brier score (95% CI)		

		Men	Women	Overall	Men	Women	Overall	Men	Women	Overall

Polypchuk et al.	PREDICT 1 equations	1.318 (1.285–1.351)	1.334 (1.291–1.377)							

	PCE	1.157 (1.112–1.202)	1.225 (1.162–1.288)							

	PCE-white									

	PCE- AA									

Sussman	VARS - CVD							0.052	0.019	

et al.	ASCVD model							0.055	0.019	

Tillin et al.	QRISK	1.20 (1.04–1.36)	1.31 (0.94–1.68)		25.7 (20.6–30.8)	29.1 (17.5–40.2)		0.12 (0.11–0.13)	0.078 (0.060–0.096)	

	FRS	1.22 (1.06–1.38)	1.30 (0.93–1.67)		26.2 (21.1–31.3)	28.7 (17.1–39.9)		0.12 (0.11–0.13)	0.079 (0.061– 0.096)	

	ASSIGN									

	QRISK2									


**Key:** ASCVD, Atherosclerotic cardiovascular disease; ASSIGN, assessing cardiovascular risk to Scottish Intercollegiate Guidelines Network to assign preventative treatment score; CVD, cardiovascular disease; FRS, Framingham risk score; PCE, Pooled Cohort Equations; QRISK, cardiovascular disease risk algorithm; VARS - CVD, Veterans Affairs Risk Score - CVD

**Table 4:** Calibration metrics

First author	Model	Predicted/Observed ratio			Other information

		Men	Women	Overall	

Bazo-Alvares et al.	FRS, Global CVD ACC/AHAWHO/ISHRRSSCORERisk chart developed by LCD GroupNon-laboratory based FraminghamPCEPooled cohort risk score				Lin’s concordance correlation coefficient (CCC); 5 geographically diverse Peruvian populations

DeFilippis	FRS-CHD	H-L = 44.75; *p* < 0.001	H-L = 30.39; *p* < 0.001		Not OE ratio Signed

et al.	FRS-CVD	H-L = 40.55; *p* = 0.001	H-L = 9.57; *p* = 0.479		Absolute difference

	ATP III-FRS-CHD	H-L = 96.02; *p* = 0.001	H-L = 15.63; *p* = 0.111		between observed

	RRS	H-L = 16.61; *p* = 0.084	H-L = 21.69; *p* = 0.017		events and predicted

	AHA/ACC/ASCVD	H-L = 62.80; *p* = 0.001	H-L = 35.98; *p* = 0.001		events (n, %)

	MESA				

	ARIC				

	RRS				

De las Heras	ACC/AHA				Calibration plot

Gala et al.	ESC SCORE				Calibration plot

Dufouil et al.	Old FSRP New FSRP				Calibration Chi-square

Fatema et al.	Model 1 FRS (laboratory based)				Net benefit fraction

	Model 2 (WHO with cholesterol)				

	Model 3 (WHO without cholesterol)				

	Model 4 FRS (non-laboratory based)				

Flueckiger et al.	R-FSRS O-FSRS PCE			2 = 6.55, *p* = 0.59 2 = 34.23, *p* = 0.0002 2 = 28.7, *p* = 0.003	Reclassification plot Net Reclassification Improvement (NRI)

Foraker et al.	CVD risk score				Calibration plot

	CVH metric				

Fox et al.	ACC/AHA CVD risk algorithm FHS				Calibration plot

Goh 2014 a et al.	Framingham risk score SCORE-Low risk score SCORE-High risk scoreSCORE		H-L = 4.74; *p* = 01918 H-L = 6.09; *p* = 0.1074 H-L = 12.06; *p* = 0.0072		

Harari et al.	SCORE-High risk				Calibration plot

	SCORE-Low risk				

	10-year risk FHS/Cox				

	20-year risk FHS/Cox				

	10-year risk Omnibus/Cox				

	20-year risk Omnibus/Cox				

Hu et al.	NEW-CHD model			H-L: before recalibration (RC) 47.7; after RC 19.7	

	FRSv2			H-L: Before recalibration (RC) 28.5; after RC 20.8	

	FRSv1			H-L: Before recalibration (RC) 173; after RC 49.0	

	FRSHDL			H-L: Before recalibration (RC) 138; after RC 25.6	

Hua et al.	Framingham 1991			10-year risk Nam-D’Agostino 2 = 82.56; *p* < 0.001	10-year risk Cook 2 = 65.91; *P* < 0.001

	Recalibrated Framingham 2008			10-year risk Nam-D’Agostino 2 = 51.09; *p* < 0.001	10-year risk Cook 2 = 34.65; *P* < 0.001

Jee et al.	Korean Heart Study Framingham CHD risk score				Net reclassification Index (NRI)

Johansson et al.	Health 2000 Finrisk				Net reclassification Index (NRI)

	Framingham				

	Reynolds				

Jung et al.	PCE White	H-L 2 = 1,364.26; *p* < 0.001	H-L 2 = 683; *p* < 0.001		

	PCE African-American	H-L 2 = 2,059.60; *p* < 0.001	H-L 2 = 604.83; *p* < 0.001		

	KRPM	H-L 2 = 25.90; *p* = 0.002	H-L 2 = 14.69; *p* < 0.100		

Kariuki et al.	Non-laboratory based Framingham	H-L 2 = 14.2; *p* = 0.115	H-L 2 = 10.5; *p* = 0.308		Calibration plot

	Laboratory-based Framingham algorithm	H-L 2 = 25.8; *p* = 0.002	H-L 2 = 21.8; *p* = 0.01		

Karjalainen	2015 SCORE Sweden			1.3; *p* = 0.12	

et al.	2003 SCORE Sweden			2.3; *p* < 0.001	

Kavousi et al.	ACC/AHA				Calibration plot

	ATP - III				

	ESC				

	PROCAM				

	RRS				

	CHD risk assessment				

Lee 2015	PCE	H-L 2 = 24.1	H-L 2 = 10.125		

et al.	Framingham CV risk equation	H-L 2 = 20.1	H-L 2 = 12.147		

	Framingham-REGIGOR				

	Trial-based approach				

	Hybrid approach				

Mortensen	US-PCE			1.2	

2017 et al.	SCORE			5.0	

Nishimura et al.	FRS and recalibrated FRS Coronary prediction algorithm for Japanese				NRI Graphical representation of H-L 2 test

Polypchuk et al.	PREDICT 1 equations PCE				Calibration plot

	ATP III				

	FRS				

Sarrafzadegan et al.	PARS risk chart	Nam-D’Agostino 2 = 24.29; *p* < 0.004	Nam-D’Agostino 2 = 7.28; *p* < 0.61		

	Framingham	Nam-D’Agostino 2 = 6.23; *p* < 0.62	Nam-D’Agostino 2 = 12.19; *p* < 0.14		

Selvarajah	FRS				

et al.	SCORE (high risk)	*p* = 0.097 graphically	*p* < 0.001		

	SCORE (low risk)	*p* = 0.067	*p* < 0.001		

	WHO/ISH				

Sun et al.	General-FRS	Modified Nam-D’Agostino 2 = 4.032; *p* < 0.402	Modified Nam-D’Agostino 2 = 9.448; *p* < 0.051		

	Simplified-FRS	Modified Nam-D’Agostino 2 = 1.160; *p* < 0.798	Modified Nam-D’Agostino 2 = 24.735; *p* < 0.001		

	SCORE-Low	Modified Nam-D’Agostino 2 = 22.430; *p* < 0.001	Modified Nam-D’Agostino 2 = 35.675; *p* < 0.001		

	SCORE-High	Modified Nam-D’Agostino 2 = 5.109; *p* < 0.276	Modified Nam-D’Agostino 2 = 28.819; *p* < 0.001		

	CN-ICVD	Modified Nam-D’Agostino 2 = 2.917; *p* < 0.405	Modified Nam-D’Agostino 2 = 5.375; *p* < 0.251		

	PCE-white	Modified Nam-D’Agostino 2 = 12.513; *p* < 0.014	Modified Nam-D’Agostino 2 = 4.572; *p* < 0.334		

	PCE-AA	Modified Nam-D’Agostino 2 = 4.032; *P* < 0.402	Modified Nam-D’Agostino 2 = 10.720; *p* < 0.030		

Sussman et al.	VARS - CVD	H-L = 17.4	23.2		

	ASCVD model	8318	49.0		

Tillin et al.	QRISK	P/O 0.75 (0.71 to 0.80)	0.74 (0.63 to 84)		

	FRS	0.99 (0.97 to 1.00)	0.70 (0.59 to 0.80)		

	ASSIGN				

	QRISK2				

Yang et al.	China-PAR	Modified Nam-D’Agostino 2 = 13.1; *p* < 0.16	Modified Nam-D’Agostino 2 = 12.8; *p* < 0.17		Calibration plot

	PCE	a) PCEs for white Americans: Modified Nam-D’Agostino 2 = 131.9; *p* < 0.001 b) PCEs for blacks: Modified Nam-D’Agostino 2 = 456.7; *p* < 0.001	a) for white Americans: Modified Nam-D’Agostino 2 = 19.4; *p* < 0.02 b) PCEs for blacks: Modified Nam-D’Agostino 2 = 77.4; *p* < 0.001		


**Key:** *AA, African-American; ACC/AHA, American College of Cardiology/American Heart Association; ARIC, Atherosclerosis Risk in Communities; ASA, American Stroke Association ; ASCVD, Atherosclerotic cardiovascular disease; ASSIGN, assessing cardiovascular risk to Scottish Intercollegiate Guidelines Network; ATP-III, Adult Treatment Panel III; CHD, coronary heart disease; CN-ICVD, Chinese ischemic CVD; China-PAR, Prediction for ASCVD Risk in China; CVD, cardiovascular disease; CVH metric, cardiovascular health metric; CVR, cardiovascular risk; ESC, European Society of Cardiology; ESC SCORE, European Society of Cardiology risk score; FHS, Framingham Heart Study; FRS, Framingham risk score; FRSHDL, HDL of FRSv1; FSRS, Framingham Stroke Risk Score; FSRP, Framingham Stroke Risk Profile; H-L, Hosmer-Lemeshow test; KRPM, Korean Risk Prediction Model; LCD, Lancet Chronic Disease; MESA, Multi-Ethnic Study of Atherosclerosis; OE ratio, observed/predicted ratio; O-FSRS, original Framingham Stroke Risk Score; PARS, Persian Atherosclerotic cardiovascular disease; PCE, Pooled Cohort Equations; PROCAM, Prospective Cardiovascular Münster score; QRISK, cardiovascular disease risk algorithm; R-FSRS, revised Framingham Stroke Risk Score; REGICOR, Registre Gironì del Cor or Girona Heart Registry; RRS, Reynolds Risk Score; SCORE, Systematic Coronary Risk Evaluation; VARS-CVD, Veterans Affairs Risk Score - CVD; WHO/ISH, World Health Organization Risk Chart*
